# A Case of Subcutaneous Panniculitis-Like T-cell Lymphoma With Hemophagocytic Lymphohistiocytosis in an HIV Patient

**DOI:** 10.7759/cureus.49564

**Published:** 2023-11-28

**Authors:** Chanakarn Kanitthamniyom, Alejandra Osorio, Sakditad Saowapa, Pharit Siladech

**Affiliations:** 1 Hematology, Thainakarin Hospital, Bangkok, THA; 2 Internal Medicine, University of Alabama at Birmingham (UAB) Hospital, Birmingham, USA; 3 Internal Medicine, Texas Tech University Health Sciences Center, Lubbock, USA; 4 Internal Medicine, Ramathibodi Hospital, Chiang Mai, THA

**Keywords:** fever of unknown origin, lupus erythematosus panniculitis, adult t cell lymphoma, painful skin lesions, cutaneous t cell lymphoma

## Abstract

Subcutaneous panniculitis-like T-cell lymphoma (SPTL) is a rare subtype of non-Hodgkin lymphoma that manifests as panniculitis-like skin lesions. It frequently co-occurs with hemophagocytic lymphohistocytosis, a life-threatening hyperinflammatory syndrome. The majority of SPTL cases express αβ T-cell receptors (SPTL-AB) and have a favorable prognosis with oral immunosuppressive agents. We report a 37-year-old male patient with HIV infection who had a history of low-grade fever for one year, multiple tender subcutaneous nodules on both thighs, and cytopenia. He received several courses of antibiotics without significant improvement. A random skin biopsy showed lobular panniculitis and he was treated with steroids, but his fever recurred after steroid withdrawal. A second skin biopsy confirmed the diagnosis of SPTL. A bone marrow examination revealed hemophagocytic lymphohistiocytosis. He was successfully treated with cyclosporin A and prednisolone and achieved a complete response after one year of drug discontinuation. Panniculitis-like skin lesions have various etiologies and may present as a clinical mimic of lupus erythematosus panniculitis. The selection of an optimal site for skin biopsy is crucial to avoid erroneous diagnoses and adverse outcomes. We report a case of SPTL in an HIV-positive patient, which illustrates this diagnostic challenge.

## Introduction

Subcutaneous panniculitis-like T-cell lymphoma (SPTL) is a rare type of T-cell lymphoma that manifests as panniculitis, a skin condition characterized by inflammation of the subcutaneous fat layer. SPTL is often associated with hemophagocytic lymphohistiocytosis (HLH), a life-threatening disorder of excessive immune activation causing hepatosplenomegaly, prolonged fever, and cytopenias [1]. SPTL is confined to the subcutaneous tissue and does not involve the lymph nodes. In 2018, the World Health Organization-European Organization for Research and Treatment of Cancer (WHO-EORTC) classified SPTL as one of the 10 subtypes of primary cutaneous T-cell lymphoma, accounting for about 1% of non-Hodgkin lymphomas [2]. The term SPTL should be reserved for cases with αβ T-cell phenotype (SPTL-AB), which can be distinguished from cases with γδ T-cell phenotype (SPTL-GD) based on immunophenotypic and prognostic differences. SPTL-AB typically expresses CD4-, CD8+, and CD56- and has a favorable outcome (five-year overall survival: 91%), whereas SPTL-GD usually expresses CD4-, CD8-, and CD56+ and has a poor prognosis (five-year overall survival: 11%) [3].

The treatment in SPTL-GD phenotype usually involves multi-agent chemotherapy, whereas SPTL-AB often responds well to oral immunosuppressive therapy.

SPTL-AB can be complicated by HLH in 15-25% of cases, which worsens the clinical course [4]. Immunocompromised patients, especially those with HIV infection, are at increased risk of developing aggressive B-cell lymphomas, such as diffuse large B-cell lymphoma (DLBCL) and Burkitt lymphoma. However, T-cell lymphomas are uncommon in this population [5]. Here, we report a case of HIV-associated SPTL-AB with HLH and describe its histopathological features and management.

## Case presentation

A 37-year-old Thai man with well-controlled HIV (compliant with HARRT, confirmed via viral suppression and a CD4 count of 158 cells/mm3) presented to a hematology clinic with a one-year history of low-grade fever and multiple subcutaneous nodules of the bilateral thighs. These skin nodules had become progressively larger and more painful and were accompanied by constitutional symptoms, including night sweats and significant weight loss (30 kg).

The patient had previously been evaluated by both general internists and infectious disease specialists and had received multiple courses of antibiotics without any significant improvement. Infectious workups including bacterial blood cultures, sputum PCR for TB, fungus, and urine culture were negative.

Subsequently, a computed tomography scan of the thorax and abdomen was executed, which showed negative lymphadenopathy and no hepatosplenomegaly. The repeat infectious workup was again negative. A random skin biopsy of the abdomen and thigh was subsequently completed. Pathology revealed lobular panniculitis, composed of various lymphocytes and histiocytes.

Laboratory evaluation demonstrated anemia and leukopenia. A bone marrow biopsy was performed. The bone marrow pathology reported mild hypocellular marrow with normal maturation. A brief course of corticosteroids was trialed on the suggestion of hematology. He defervesced on steroids but when the steroids were discontinued, he again became febrile.

At his visit to my clinic, the patient was febrile to 38.2°C. On examination, both thighs revealed multiple discrete skin indurations that were painful and did not have surrounding erythema. Laboratory results showed cytopenia, with a white blood cell count of 1570 cells/mcL (neutrophils 79%, lymphocytes 9%, monocytes 4%), hemoglobin of 8.4 g/dL, and platelets of 278,000 plts/mcL. The patient was found to have a conspicuously elevated ferritin level of 13,843 ng/mL (normal range 30-400 ng/mL), a fibrinogen level of 77 mg/dL (normal range 180-350 mg/dL), and a triglyceride level of 350 mg/dL (normal range <150 mg/dL). Serologic results for hepatitis including HBV and HCV, EBV, and CMV were negative.

**Figure 1:**
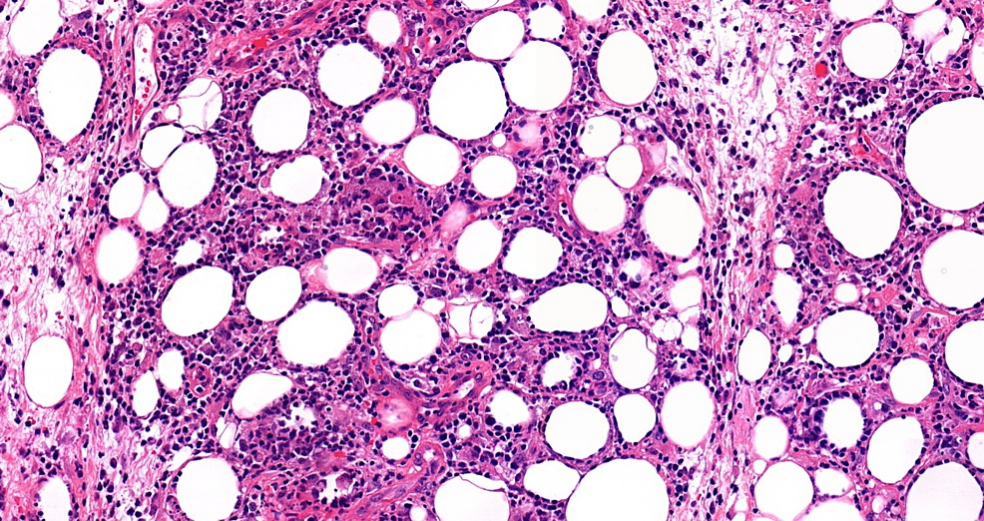
Rimming of the adipocytes by atypical lymphocytes (hematoxylin and eosin stain)

**Figure 2:**
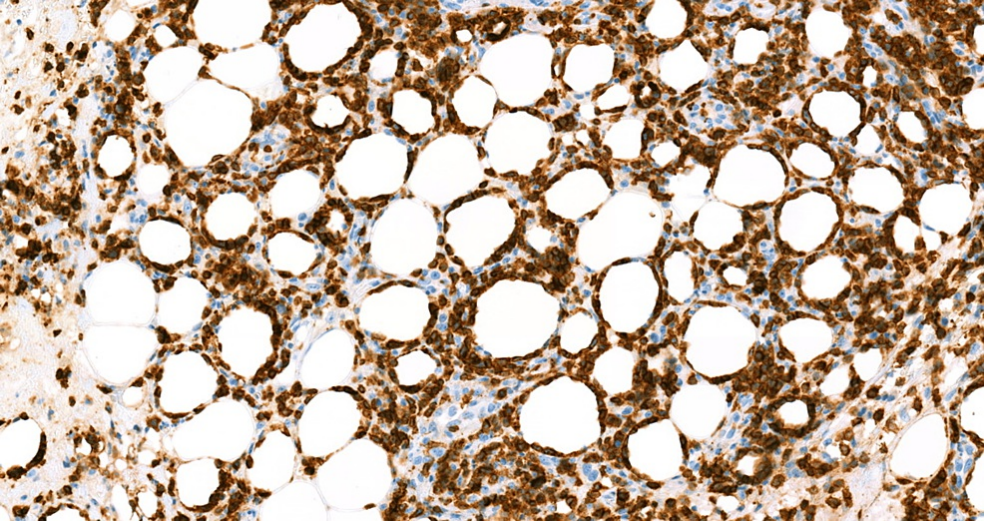
CD3 immunohistochemical stain of the atypical lymphocytes, demonstrating T-cell lineage

**Figure 3:**
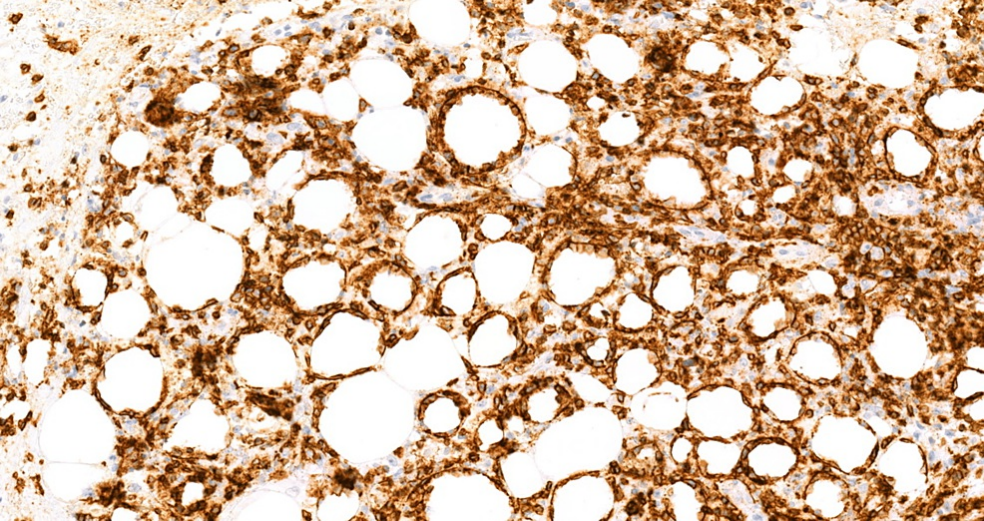
CD8 immunohistochemical stain highlights the atypical lymphocytes, also demonstrating T-cell lineage

**Figure 4:**
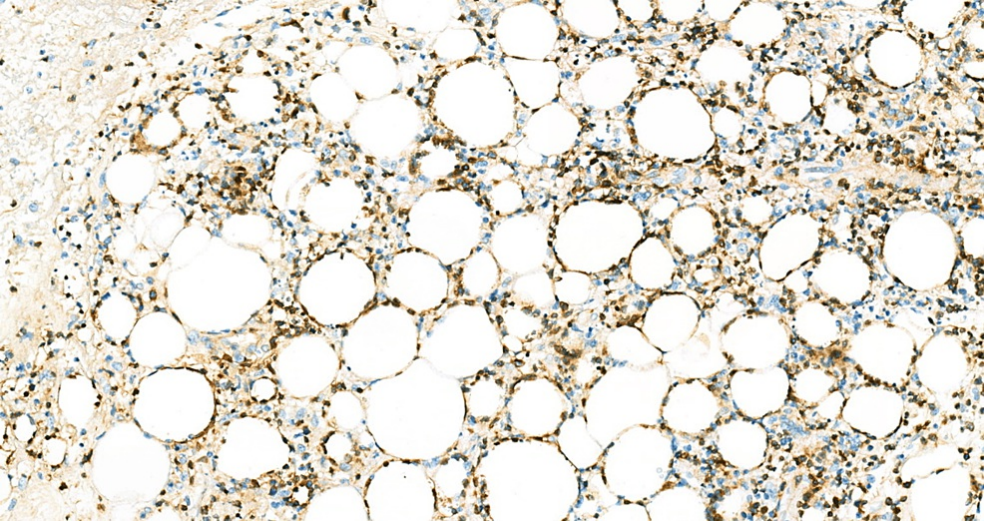
Beta-F1 immunohistochemical stain highlights the atypical lymphocytes

The patient's laboratory results were consistent with secondary HLH with concern for possible cutaneous lymphoma. Lymphoma-specific flow cytometry yielded negative results. A repeat excisional skin biopsy at the indurated areas and a repeat bone marrow study were performed. Immunohistochemical analysis of the skin biopsy showed positive expression of CD3, CD8, TIA-1, and BF1 in cytotoxic T-cells, predominantly surrounding adipocytes, and negative expression of GTCR, CD56, and CD4 compatible with SPTL (Figures 1-4).

**Figure 5:**
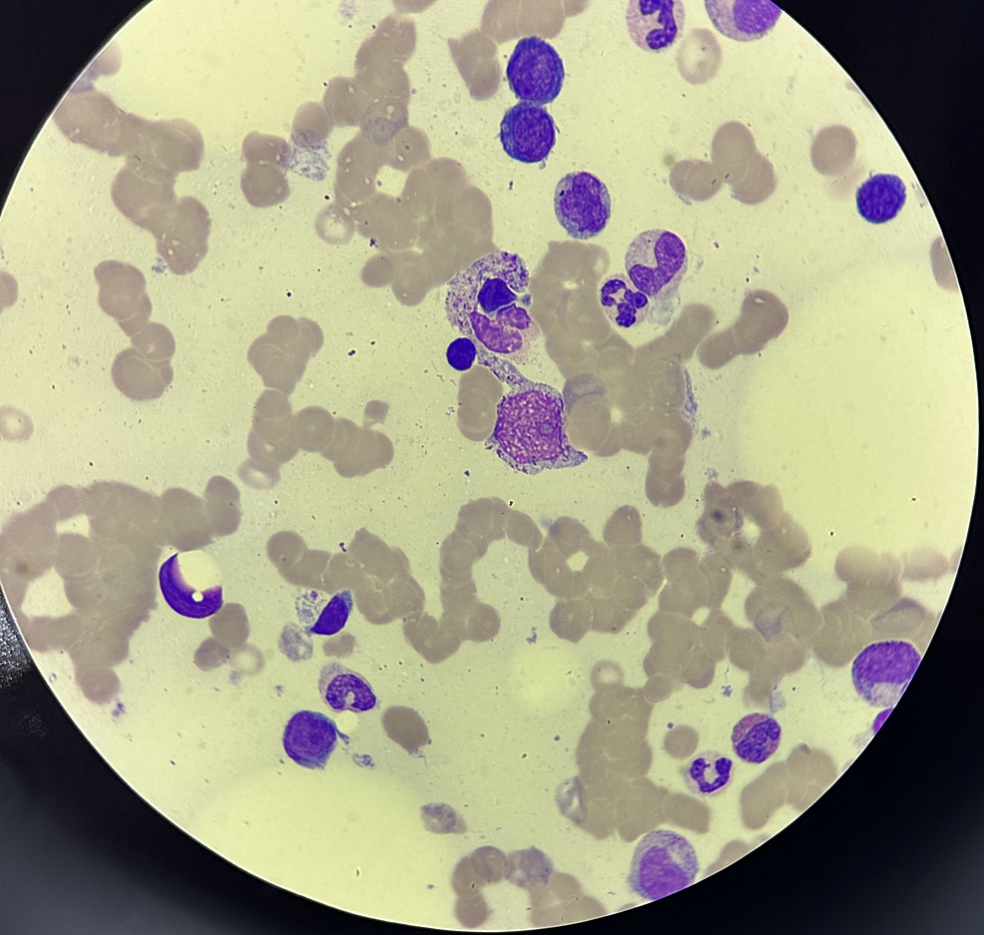
Bone marrow smear showing an increase in hemophagocytic activity

In addition, the repeat bone marrow smear demonstrated hemophagocytic histiocytes with normocellular with trilineage hematopoiesis (Figure 5).

As a consequence of these cumulative findings, the patient was diagnosed with SPTL with secondary HLH. He was placed on cyclosporine A 200 mg/d and prednisolone 50 mg/d (1MKD).

The patient's condition improved after one month of hematology clinic follow-up. Resolution of fever transpired alongside weight restoration. His laboratory tests also showed normalization. His prednisolone dose was gradually reduced over four months and he remained on maintenance cyclosporine A for one year. After one year, cyclosporine A was discontinued without symptom recurrence.

## Discussion

This particular case of SPTL was diagnosed late after multiple courses of antibiotics and a misdiagnosis of lupus panniculitis. The diagnosis of SPTL can be challenging, especially in patients with HIV infection, for several reasons. First, fever in HIV patients can have various etiologies, such as opportunistic infections, autoimmune complications, or HIV itself. Second, the most common lymphomas in HIV patients are of B-cell origin such as DLBCL or Burkitt lymphoma; thus, SPTL is not a likely differential diagnosis for most physicians. Lastly, SPTL can mimic several other conditions that present with subcutaneous nodules, such as lupus erythematosus panniculitis, systemic vasculitis, erythema nodosum, and lymphoid hyperplasia [6,7]. Therefore, a skin biopsy is a crucial step in establishing the diagnosis. Given the unique histopathology surrounding SPTL, skin biopsies should be reviewed by an experienced hematopathologist in order to correctly identify the disease. Additionally, selecting an insignificant skin lesion can lead to a false-negative result, as in our case. Repeated biopsies are often required to yield a definitive diagnosis in patients with SPTL. Fluorodeoxyglucose positron emission tomography (FDG-PET) is a useful and sensitive tool to detect all sites of cutaneous and extracutaneous involvement of lymphoma but was unable to be performed in this case. However, PET imaging can guide the choice of a skin lesion for biopsy based on the degree of metabolic activity and increase diagnostic yield [6].

Various treatment options are available for SPTL, such as radiotherapy, immunosuppressive therapy, and chemotherapy. Multi-agent chemotherapy with anthracycline-based chemotherapy is often employed in patients with aggressive diseases. However, we opted to treat with cyclosporine and steroids based on various other case reports demonstrating rapid response to treatment and sustained remission [8]. Thus far, the patient has exhibited favorable clinical and laboratory outcomes.

## Conclusions

We report a case of an HIV-positive patient who was diagnosed late with SPTL and developed HLH. This case highlights the importance of considering other diagnoses in patients who do not respond well to previous treatment and selecting an appropriate site for a skin biopsy to avoid misdiagnosis. The patient responded well to cyclosporine with a steroid-containing regimen and achieved a sustained response after discontinuing treatment.
